# Assessment of Scleral and Conjunctival Thickness of the Eye after Ultrasound Ciliary Plasty

**DOI:** 10.1155/2020/9659014

**Published:** 2020-09-24

**Authors:** Bartłomiej Bolek, Adam Wylęgała, Edward Wylęgała

**Affiliations:** Chair and Clinical Department of Ophthalmology, School of Medicine in Zabrze, Medical University of Silesia in Katowice, District Railway Hospital, Katowice, Poland

## Abstract

**Purpose:**

This study aims to assess scleral and conjunctival thickness using optical coherence tomography after ultrasound ciliary plasty (UCP) procedure with reference to scleral marks appearing in the area where the ultrasound energy was applied.

**Materials and Methods:**

Seventy-eight patients with primary and secondary refractory glaucoma participated in this study. Complete ophthalmic examinations including measurements of scleral and conjunctival thickness were performed preoperatively and at 1 week, and 1, 3, 6, 12, 18, and 24 months postoperatively. The parameters were determined using the Swept Source OCT with anterior attachment. Thirty-eight patients (58 scleral marks—23 superior and 35 inferior) fulfilled the inclusion criteria and completed the follow-up period of 24 months.

**Results:**

The mean ± SD scleral and conjunctival thickness in superior scleral mark before the procedure and at 1 week, and 1, 3, 6, 12, 18, and 24 months after the procedure was 684.57 ± 83.58 *μ*m, 771.78 ± 112.03 *μ*m (*p* < 0.001), 771.74 ± 100.12 *μ*m (*p* < 0.001), 731.38 ± 83.92 *μ*m (*p*=0.012), 719.52 ± 73.20 *μ*m (*p*=0.037), 702.91 ± 66.50 *μ*m (*p*=0.247), 694.13 ± 72.22 *μ*m (*p*=0.482), and 699.35 ± 70.68 *μ*m (*p*=0.200), respectively. The mean ± SD scleral and conjunctival thickness in inferior scleral mark before the procedure and at 1 week, and 1, 3, 6, 12, 18, and 24 months after the procedure was 816.86 ± 79.30 *μ*m, 936.37 ± 107.33 *μ*m (*p* < 0.001), 946.00 ± 130.40 *μ*m (*p* < 0.001), 896.63 ± 123.40 *μ*m (*p* < 0.001), 877.69 ± 114.38 *μ*m (*p*=0.003), 843.03 ± 71.55 *μ*m (*p*=0.021), 811.86 ± 68.91 *μ*m (*p*=0.731), and 805.03 ± 69.52 *μ*m (*p*=0.248), respectively. The transient thickening of the sclera was observed after the procedure; however, after 12 months postoperatively, the parameters returned to the initial value and no significant difference was noted.

**Conclusion:**

The sclera thickness increases after UCP. However, with time the thickness reduces to its initial value with no significant difference. Clinical implication of the scleral changes lasts shorter than the measured significant difference in scleral thickness.

## 1. Introduction

Cyclodestruction methods are used to treat mild and severe forms of glaucoma. These methods reduce intraocular pressure (IOP) by decreasing the production of aqueous humor by partially damaging the nonpigmented epithelium of the ciliary body. Ultrasound ciliary plasty (UCP), compared to commonly used diode laser cyclodestruction, allows precise energy concentration through opaque structures, without uncontrolled absorption, at a desired depth and area [1]. As a result, it reduces the damage to adjacent tissues. However, ultrasound energy may affect sclera, leading to an alteration in morphology and morphometry. In the area where the ultrasound energy was applied, a scleral mark occurs, which macroscopically appears like scleral thinning. Few studies have reported the occurrence of scleral marks but without further examination [2–5]. A study conducted by Mastropasqua et al. showed anatomical modifications of sclera and conjunctiva in one month after UCP [6]. The authors assessed cyst occurrence in the area of ultrasound energy application intending to prove alternative outflow of aqueous humor through the uveoscleral pathway. There is no study examining scleral mark in terms of scleral thinning after UCP. The present study aims to assess scleral and conjunctival thickness using optical coherence tomography (OCT) after UCP with reference to scleral marks appearing in the area of ultrasound energy application in the long-term period.

## 2. Materials and Methods

This retrospective clinical study was approved by the institutional review board of the Medical University of Silesia (KNW/0022/KB1/78/18). Considering that the study involved a retrospective review of existing data, specific written informed consent was obtained from all patients. However, informed consent regarding the UCP was obtained from the patients who received the procedure.

Seventy-eight patients with primary and secondary refractory glaucoma were enrolled to undergo UCP. The inclusion criteria for the study were: adult patients (≥18 years), uncontrolled glaucoma (IOP > 21 mmHg, despite the maximum tolerated doses of antiglaucoma medications), and intolerance to glaucoma medications despite well-controlled IOP. The exclusion criteria were as follows: patients aged <18 years, IOP >30 mmHg, neovascular glaucoma, scleral mark not visible after the procedure, poor-quality OCT scan, and previous glaucoma surgeries involving perilimbal interference in the sclera. Thirty-eight patients (58 scleral marks—23 superior and 35 inferior) fulfilled the abovementioned criteria and completed the 24-month follow-up period.

**Figure 1:**
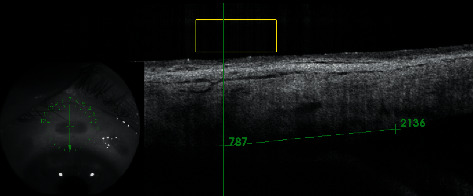
OCT scan of a patient three months after UCP—distance from limbus to scleral mark was measured (2136 *μ*m) to determine preoperative thickness.

**Figure 2:**
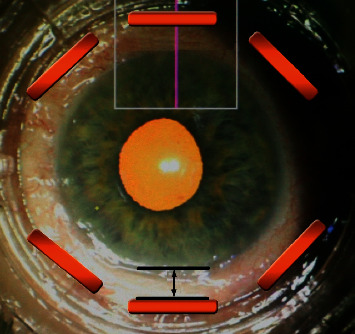
Scheme of the scleral and conjunctival measurements after UCP. OCT scans were taken in a projection perpendicular to the limbus of the cornea in the superior and inferior quadrants of the eye (purple continuous line). Thickness values were measured equidistant from the limbus (black continuous line) where the scleral mark occurred (orange rectangle).

**Figure 3:**
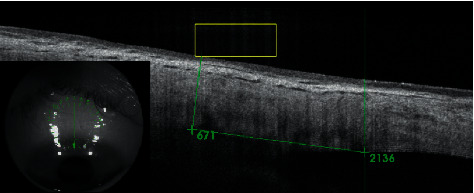
OCT scan of a patient before UCP—distance from limbus to the point where sclera and conjunctiva thickness measurements should be taken (2136 *μ*m) was derived from the scan obtained after the procedure (Figure 1).

Complete ophthalmic examinations with measurements of sclera and conjunctiva thickness (*μ*m) were performed preoperatively and at 1 week, and 1, 3, 6, 12, 18, and 24 months after the procedure. The parameters were determined using Swept Source OCT with anterior attachment (DRI OCT Triton, Topcon Inc., Tokyo, Japan). Scleral and conjunctival thickness was measured manually, by two operators (BB, AW), using the built-in measuring tool in the OCT device. The mean of both measurements was taken as a result of the analysis. The scleral OCT scans were obtained in a projection perpendicular to the limbus of the cornea in the superior and inferior quadrants of the eye. The thickness was measured in the center of the area where the scleral mark occurred (Figures 1 and 2). Preoperative thickness was measured equidistant from limbus to scleral mark, and the accuracy of the measurement was determined from the control scans obtained after the procedure where scleral marks were visible (Figure 3). The first high reflective tissue signal was considered to be the outer limit of the scleral and conjunctival thickness, and the interface between the sclera (highly reflective) and choroid (less reflective) was considered the inner limit (Figures 1 and 3). Eyes without scleral mark after the procedure were excluded from the study as the exact area of ultrasound energy application could not be determined.

Additionally, IOP (determined using Goldmann applanation tonometer) and the number of antiglaucoma medications were included in the analysis. An IOP reduction of 20% or >5 mmHg as compared to the baseline value was considered as an indication of successful treatment. Complete success was defined as the cessation of antiglaucoma medications.

The UCP procedure was performed using the EyeOP1 device (Eye Tech Care, Rillieux-la-Pape, France) under intravenous or peribulbar anesthesia. We used a second-generation ring-shaped probe containing six piezoelectric components (transducers) with a high frequency of 21 MHz (high-intensity focused ultrasound—HIFU technology). The probe size was determined based on the axial length and white-to-white parameters measured before surgery using IOL Master 700 (Carl Zeiss, Meditec AG, Jena, Germany). The procedure involved precise adjustment of positioning cone at the center of the eye under a surgical microscope, stabilized by a mild vacuum system. The probe was then inserted into the cone and transducers were sequentially activated by footswitch. Each of the six transducers had an operation time of 8 s with a 20 s interval between the subsequent exposures which made it an approximately 3 min procedure in total (second-generation probe). The preoperative antiglaucoma medications were either continued as before or modified according to the postoperative IOP. Postoperatively, patients were treated topically with ofloxacin (five times a day for two weeks), dexamethasone (five times a day for two weeks, followed by three times a day for two weeks), and atropine (three times a day for two weeks).

Statistical analysis was performed using Statistica Version 13 (TIBCO Software Inc., Palo Alto, CA). Groups of data sets for a given parameter were compared using the Wilcoxon signed-rank test or *t*-test depending on the data distribution. Spearman's correlation analysis was used to investigate the relations between scleral and conjunctival thickness and IOP. A *p* value of 0.05 or less was considered to represent statistical significance.

## 3. Results

The results are presented for 38 patients (58 scleral marks-23 superior and 35 inferior) during a 2-year follow-up. The patient characteristics are described in Table 1.

The mean ± SD scleral and conjunctival thickness in superior scleral mark before the procedure and at 1 week, and 1, 3, 6, 12, 18, and 24 months after the procedure was 684.57 ± 83.58 *μ*m, 771.78 ± 112.03 *μ*m (*p* < 0.001), 771.74 ± 100.12 *μ*m (*p* < 0.001), 731.38 ± 83.92 *μ*m (*p*=0.012), 719.52 ± 73.20 *μ*m (*p*=0.037), 702.91 ± 66.50 *μ*m (*p*=0.247), 694.13 ± 72.22 *μ*m (*p*=0.482), and 699.35 ± 70.68 *μ*m (*p*=0.200), respectively (Table 2).

The mean ± SD scleral and conjunctival thickness in inferior scleral mark before the procedure and at 1 week, and 1, 3, 6, 12, 18, and 24 months after the procedure was 816.86 ± 79.30 *μ*m, 936.37 ± 107.33 *μ*m (*p* < 0.001), 946.00 ± 130.40 *μ*m (*p* < 0.001), 896.63 ± 123.40 *μ*m (*p* < 0.001), 877.69 ± 114.38 *μ*m (*p*=0.003), 843.03 ± 71.55 *μ*m (*p*=0.021), 811.86 ± 68.91 *μ*m (*p*=0.731), and 805.03 ± 69.52 *μ*m (*p*=0.248), respectively (Table 2).

**Figure 4:**
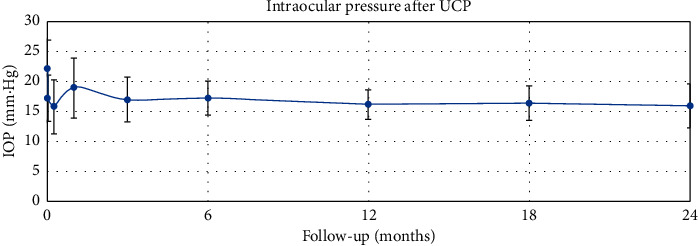
Intraocular pressure after UCP—24-month follow-up.

The mean ± SD values of IOP measured preoperatively and at 1 week, and 1, 3, 6, 12, 18 and 24 months postoperatively was 22.2 ± 4.7 mmHg, 15.8 ± 4.5 mmHg (*p* < 0.001), 18.9 ± 5.0 mmHg (*p* < 0.001), 17.0 ± 3.7 mmHg (*p* < 0.001), 17.3 ± 2.9 mmHg (*p* < 0.001), 16.2 ± 2.5 mmHg (*p* < 0.001), 16.4 ± 2.8 mmHg (*p* < 0.001), and 16.0 ± 3.6 mmHg (*p* < 0.001), respectively (Table 3, Figure 4). The mean IOP at the last follow-up was reduced by 28.1% (Table 3). The success rate and the complete success rate were 89.7% and 7.7%, respectively.

**Figure 5:**
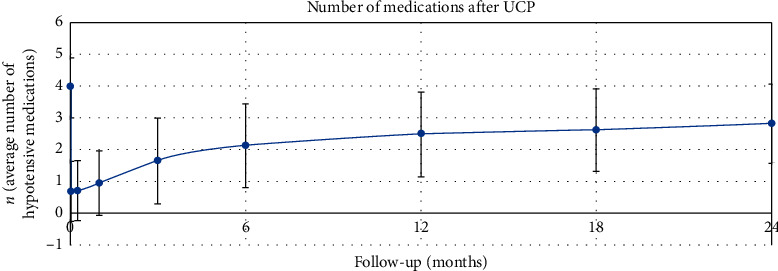
The number of antiglaucoma medications after UCP—24-month follow-up.

The mean ± SD number of antiglaucoma medications preoperatively and at 1 week, and 1, 3, 6, 12, 18 and 24 months postoperatively was 4.0 ± 0.8, 0.7 ± 0.9 (*p* < 0.001), 0.9 ± 1.0 (*p* < 0.001), 1.6 ± 1.3 (*p* < 0.001), 2.1 ± 1.3 (*p* < 0.001), 2.5 ± 1.3 (*p* < 0.001), 2.6 ± 1.3 (*p* < 0.001), and 2.8 ± 1.3 (*p* < 0.001), respectively (Table 3, Figure 5).

Correlation analysis revealed no correlation between scleral and conjunctival thickness in superior or inferior scleral mark and IOP. The results are presented in Table 4.

Choroid detachment was observed in one patient (2.6%), and macular edema was also observed in one patient (2.6%). No other major intraoperative or postoperative complications occurred.

**Figure 6:**
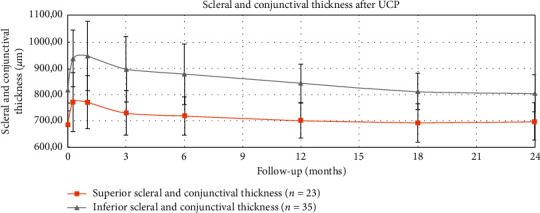
Scleral and conjunctival thickness in superior and inferior scleral mark after UCP—24-month follow-up.

The results revealed a significant difference in the scleral and conjunctival thickness after the UCP procedure. However, after 12 months, the parameters returned to the initial values with no significant difference (Figure 6). The decrease in IOP and the number of antiglaucoma medications used were statistically significant (Table 3). There is no correlation between scleral and conjunctival thickness and IOP.

## 4. Discussion

Currently, the only effective and proven method to treat glaucoma is to reduce IOP [7, 8]. This can be achieved by limiting the production of aqueous humor and/or improving its outflow with pharmacological and surgical methods. Production of aqueous humor can be reduced by partially damaging the nonpigmented epithelium of the ciliary body using laser photocoagulation, cryotherapy, or ultrasound energy. The most common and effective method used for this is transscleral cyclophotocoagulation (TSCP). TSCP is mainly used in severe cases of refractory glaucoma, when previous pharmacological or surgical (filtration or seton) treatment was not successful [9]. Two main disadvantages of cyclodestruction are the limited selectivity of target tissue often which causes damage to adjacent structures (e.g., laser energy is primarily absorbed by pigmented tissues, damaging the iris or choroid) and difficult prediction of the effect in relation to the dose used. TSCP also carries a risk of complications. The most common consequences are pain during and after surgery, conjunctival burn, sclera thinning, and uveitis [10–15]. Rare, but more serious, complications are hypotension, choroidal detachment, choroiditis, retinal detachment, or extremely rare-phthisis bulbi [16]. Endoscopic cyclodestruction (ECP) is better in terms of safety and selectivity compared to TSCP [17, 18]. However, it is an invasive procedure and is recommended only for mild or moderate glaucoma patients undergoing cataract surgery [19, 20]. This procedure also has side effects, and the most common ones are IOP spikes, increased inflammation (compared to phacoemulsification without ECP), and dislocation of the intraocular lens [21, 22].

Compared to the commonly used diode laser cyclodestruction, the main advantage of the HIFU technology, used in UCP and through a specially designed probe is the possibility of achieving precise energy concentration through opaque structures, without uncontrolled absorption, at a desired depth and area of the ciliary body [1]. As a result, it reduces the damage to adjacent tissues because the amount of heat delivered to the tissue does not depend on its properties, for example, pigmentation, which in the case of the ciliary body may vary from person to person [23–25]. In spite of these advantages, the exact influence of ultrasound energy on the conjunctival and scleral tissues, and indirectly on the cornea, is still unknown.

Although a few studies have reported the appearance of scleral marks after UCP, their authors did not specify those changes consistently. Denis et al. reported scleral thinning without any sign of inflammation, induced corneal astigmatism, or scleral protrusion [3]. Deb-Joardar et al. described these findings as probable focal shrinkage of scleral tissue [2]. On the contrary, a meta-analysis of the UCP procedure reported no scleral thinning observed on OCT; however, it is not supported by any research [4]. The abovementioned studies focused mainly on the efficiency of the procedure without any further examination of the occurrence of scleral marks. These were later analyzed by two studies. The first one conducted by Mastropasqua et al. was designed to assess the alternative outflow of aqueous humor through the uveoscleral pathway using anterior segment OCT (AS-OCT) and confocal microscopy [6]. The authors reported the occurrence of a sclera cyst in the area where the ultrasound energy was applied and attributed it to heat-induced scleral fiber delamination. They did not find any scleral thinning in AS-OCT but could not rule out that occurrence. The second study was conducted on pigs' eyes and histologically reported scleral marks as rearrangement of the tissue. It was observed that collagen presented different elasticity, resistance, permeability, and opacity properties resulting in a more compact and denser structure. Its refractive power differed from that of a healthy area which may explain the translucid and grayish appearance found macroscopically [26].

Not all patients present scleral marks after the UCP procedure. Only two papers reported the exact appearance of scleral marks in treated patients—Deb-Joardar et al. in six out of 28 patients (21%) [2] and De Gregorio et al., in 10 out of 40 of patients (25%). [27] In both reports, a second-generation probe with 8 s exposure time was used. Two more papers reported scleral thinning in one out of 30 patients [28] and two out of 28 patients [29], respectively, and although the findings were not described as scleral marks, we can assume it as the same phenomenon. However, here, the first-generation probe with 6 s exposure time was used. All of the above studies did not explain how the findings were examined. A meta-analysis of the UCP procedure [4] reported that scleral marks were more common in Indian eyes than in Caucasian eyes, which can be attributed to the more pigmented sclera. It also indicated that there was variation in the pattern of scleral mark development: in some cases, the marks faded over time, whereas in others, the reverse occurred. In our study group (78 patients), the rate of occurrence of visible scleral marks was 77.4%. Undoubtedly, the exposure time of ultrasound energy has an influence on the frequent appearance of scleral marks. However, there is a difference between our results and that of the first two studies, which used the same generation probe, with a predominance of scleral mark occurrence in our study. It is hard to explain the difference since the amount of energy delivered by the same generation probe is always constant. Scleral marks differ in intensity. We examined our patients very thoroughly, looking for scleral marks. Even slightly visible scleral mark was reported. The two cited studies focused mainly on UCP efficacy. The authors reported the scleral mark as a complication and did not explain how the occurrence was examined. Only well visible scleral marks were possibly taken to analysis which could have caused the discrepancies.

**Figure 7:**
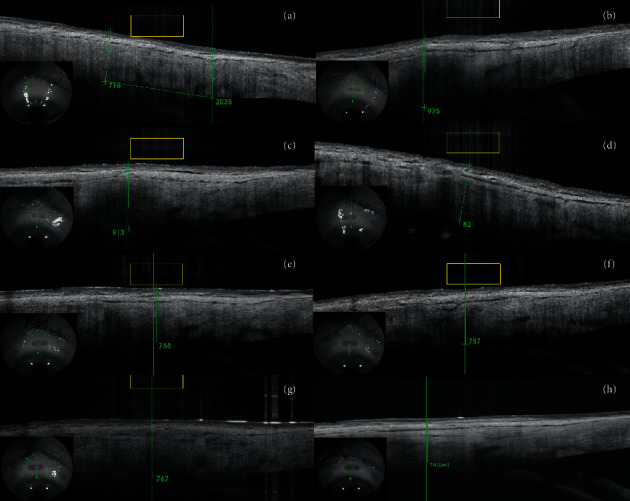
Superior-quadrant OCT scans of the sclera and conjunctiva of a patient before the procedure (a), at 1 week (b), and 1 (c), 3 (d), 6 (e), 12 (f), 18 (g), and 24 (h) months after UCP—24-month follow-up period.

**Figure 8:**
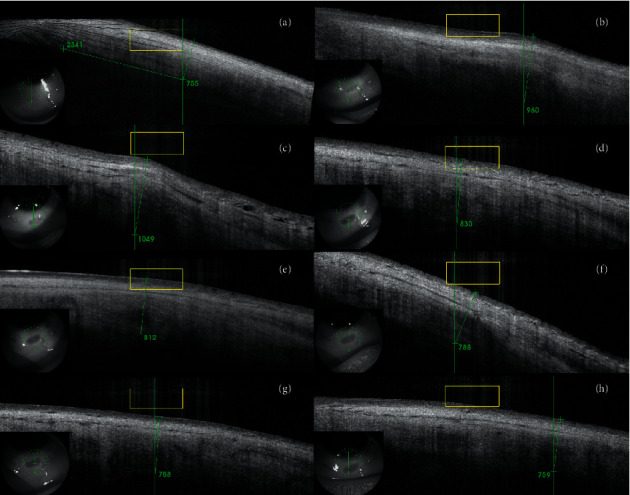
Inferior-quadrant OCT scans of the sclera and conjunctiva of a patient before the procedure (a), at 1 week (b), and 1 (c), 3 (d), 6 (e), 12 (f), 18 (g), and 24 (h) months after UCP—24-month follow-up period.

Macroscopically, in a slit-lamp examination, scleral marks look like scleral thinning which was the reason for conducting this study. However, after measuring the sclera and conjunctiva, we did not observe thinning in any of our patients. On the contrary, there was a transient thickening (Figures 7 and 8). The measured parameters returned to initial values 18 months after the UCP procedure (with no significant difference). The OCT scans showed more indistinct hyporeflective scleral tissue in the area where the ultrasound energy was applied. The conjunctiva was intact and changes were visible only in the sclera. However, we did not observe delineated intrascleral hyporeflective spaces as reported by Mastropasqua et al. [6] or scleral protrusion.

Postoperatively, the patients were treated topically with dexamethasone for one month. The application of this anti-inflammatory therapy after UCP can have an influence in terms of conjunctival inflammation or scleral edema. However, dexamethasone was given only during the first month, yet the follow-up period of the study was 2 years. As it is a standard postoperative treatment after any cyclodestruction procedure, skipping it would lead to negative consequences.

IOP >30 mmHg and neovascular glaucoma were exclusion criteria of our study. Studies on UCP revealed that efficacy in these cases might be unsatisfactory after one treatment [5, 27]. The manufacturer of the UCP device also does not recommend using this type of procedure and six-sector protocol for treating neovascular glaucoma in patients with IOP >30 mmHg. Instead, in such patients, the manufacturer recommends using eight-sector protocol—two additional sectors of the ciliary body are treated. At the beginning of the study, a UCP Flex probe (Eye Tech Care, Rillieux-la-Pape, France) with eight-sector protocol was not yet available.

Previous glaucoma surgeries, like trabeculectomy or TSCP, directly or indirectly involving perilimbal interference in the sclera, were another exclusion criterion of our study. In the scanned area, the sclera needs to be intact to reliably analyze and measure its thickness.

The decrease in IOP and the number of antiglaucoma medications was statistically significant at each follow-up time point. The mean IOP at the last follow-up was reduced by 28.1%, which is similar to other studies [2, 3, 28, 30]. Correlation analysis revealed no correlation between scleral and conjunctival thickness and IOP.

Choroid detachment was observed in one patient (2.6%) in one week after the procedure. This complication resolves in one month after discontinuation of antiglaucoma medications. Macular edema occurred in one patient (2.6%) one month after the procedure, and this patient had coexistence of the epiretinal membrane. Edema resolves in two months after pharmacotherapy-with topical nonsteroidal anti-inflammatory drug and oral carbonic anhydrase inhibitor. No other major intraoperative or postoperative complications, such as cataract, retinal detachment, or phthisis bulbi, were observed.

The present study has some limitations. First, it is not possible to use OCT to precisely distinguish the boundary of sclera and conjunctiva. This was the reason we measured the thickness of both sclera and conjunctiva in the area of energy application. The OCT scans showed the conjunctiva to be intact and changes in tissues after UCP were visible only in the sclera. Second, OCT scans have limitations in the case of scleral tissue penetration. Not in every scan we can see a clear boundary between the sclera and the choroid. For analysis, we selected those OCT scans in which we could precisely distinguish the scleral boundaries. We did not investigate the choroid because we could not image this site easily using OCT. In our experience, AS-OCT (Casia 2 Swept Source OCT, Tomey, Tokyo, Japan) has lower scleral penetration than Swept Source OCT dedicated to the posterior segment with an anterior segment adapter, despite the best possible manual scan settings. Therefore, a second type of equipment was used. Despite these imperfections, AS-OCT provides more accurate delineation of the anterior segment structures with higher scanning resolution than ultrasound biomicroscopy [31, 32]. Third, the measurement of OCT thickness may present a level of subjectivity. All measurements were performed manually by two operators using the built-in measuring tool in the OCT device. Fourth, in our study, we analyzed the most upper and lower scleral marks. We did not analyze the other four scleral marks (in upper/lower nasal/temporal quadrant). To obtain the OCT scan of these areas, the patient should look in the opposite direction to the scleral mark. We noticed that in this case, patient's cooperation is worse than looking straight up or down. This resulted in poor-quality OCT scans which were difficult to measure. However, we found no apparent differences in the sclera of those areas compared to the analyzed one.

Despite the assumptions in reducing the energy applied to neighboring tissues in the UCP procedure, the literature and our study show that this cyclodestruction method affects the sclera. However, the effect was transient, and we did not observe scleral thinning or protrusion. On the contrary, there was a temporary thickening. Also, we did not observe any correlation between changes in scleral thickness and IOP. Clinically, changes in the sclera may lead to the appearance of astigmatism after the UCP procedure. In another study conducted by our clinic, we proved that UCP affects corneal topography immediately after the procedure (mostly anterior astigmatism). However, during due the course of time (after three months), the corneal parameters return to initial values [33]. Therefore, clinical implication of changes lasts shorter than a significant difference in scleral thickness. This information shows that there are no contraindications in the profile of patients suitable for this type of surgery in terms of scleral abnormalities. It will also allow safe treatment of refractory glaucoma—the advanced stage of the disease, where pharmacological treatments have already been taken, and this type of surgery is the only way to stop the process of vision loss.

## 5. Conclusion

Scleral thickness changes after the UCP procedure. However, with time, the parameter returns to its initial value. Clinical implication of sclera changes lasts shorter than the significant measured difference in scleral thickness.

## Figures and Tables

**Table 1:** Demographic characteristics

Demographic characteristics	
Age (years), mean (SD) [range]	67.56 ± 11.63[26–81]
Gender (male/female)	24/14
Type of glaucoma	
Primary open-angle glaucoma	32
Secondary open-angle glaucoma	
Postpenetrating keratoplasty glaucoma	4
Exfoliative	2
Previous glaucoma treatements	
Endocyclophotocoagulation	1
SLT	4
Lens status	
Phakic	20
Pseudophakic	17
Aphakic	1

SD: standard deviation.

**Table 2:** Scleral and conjunctival thickness in superior and inferior scleral mark after UCP—24-month follow-up.

Mean values ± SD (*p* value)	Preop	1 week	*p* value	1 month	*p* value	3 months	*p* value	6 months	*p* value	12 months	*p* value	18 months	*p* value	24 months	*p* value
Superior scleral and conjunctival thickness (*µ*m)	684.57 ± 83.58	771.78 ± 112.03	<0.001	771.74 ± 100.12	<0.001	731.48 ± 83.92	0.012	719.52 ± 73.20	0.037	702.91 ± 66.50	0.247	694.13 ± 72.22	0.482	699.35 ± 70.68	0.200
Inferior scleral and conjunctival thickness (*µ*m)	816.86 ± 79.30	936.37 ± 107.33	<0.001	946.00 ± 130.40	<0.001	896.63 ± 123.40	<0.001	877.69 ± 114.38	0.003	843.03 ± 71.55	0.021	811.86 ± 68.91	0.731	805.03 ± 69.52	0.248

SD: standard deviation.

**Table 3:** Intraocular pressure and number of hypotensive medications after UCP—24-month follow-up

Mean IOP ± SD		*p* value	Number of hypotensive medications SD	*p* value	% IOP reduction
Preop	22.2 ± 4.7		4.0 ± 0.8		—
1 week	15.8 ± 4.5	*p* < 0.001	0.7 ± 0.9	*p* < 0.001	28.8
1 month	18.9 ± 5.0	*p* < 0.001	0.9 ± 1.0	*p* < 0.001	14.8
3 months	17.0 ± 3.7	*p* < 0.001	1.6 ± 1.3	*p* < 0.001	23.3
6 months	17.3 ± 2.9	*p* < 0.001	2.1 ± 1.3	*p* < 0.001	22.3
12 months	16.2 ± 2.5	*p* < 0.001	2.5 ± 1.3	*p* < 0.001	27.0
18 months	16.4 ± 2.8	*p* < 0.001	2.6 ± 1.3	*p* < 0.001	26.0
24 months	16.0 ± 3.6	*p* < 0.001	2.8 ± 1.3	*p* < 0.001	28.1

UCP: ultrasound ciliary plasty; IOP: intraocular pressure; SD: standard deviation.

**Table 4:** Correlation between scleral and conjunctival thickness in superior/inferior scleral mark and IOP after UCP.

SCT versus IOP	Preop		1 week		1 month		3 months		6 months		12 months		18 months		24 months	
	*r*	*p* value	*r*	*p* value	*r*	*p* value	*r*	*p* value	*r*	*p* value	*r*	*p* value	*r*	*p* value	*r*	*p* value
Superior SCT vs. IOP	0.186	0.395	0.343	0.109	0.153	0.487	0.032	0.883	−0.082	0.710	0.143	0.516	0.149	0.497	0.092	0.677
Inferior SCT vs. IOP	0.061	0.727	0.044	0.804	−0.099	0.570	0.003	0.985	0.119	0.497	0.225	0.195	0.300	0.080	0.120	0.491

SCT: scleral and conjunctival thickness; IOP: intraocular pressure.

## Data Availability

The numerical data used to support the findings of this study may be released upon application to the Chair and Clinical Department of Ophthalmology, School of Medicine in Zabrze, the Medical University of Silesia in Katowice, District Railway Hospital, Katowice, Poland, who can be contacted at District Railway Hospital, Panewnicka 65, 40–760 Katowice, Poland; e-mail: bartlomiej.bolek@med.sum.edu.pl;

## Abbreviations

UCP: Ultrasound cliary plasty
SS-OCT: Swept source optical coherence tomography
TSCP: Transscleral cyclophotocoagulation
SIA: Surgical induced astigmatism
IOP: Intraocular pressure
AXL: Axial length
WTW: White to white
HIFU: High-intensity focused ultrasound.
